# A Novel View of the Diversity of Anoxygenic Phototrophic Bacteria Inhabiting the Chemocline of Meromictic Karst Lakes

**DOI:** 10.3390/microorganisms12010013

**Published:** 2023-12-20

**Authors:** Vladimir Gorlenko, Alexander Savvichev, Vitaly Kadnikov, Igor Rusanov, Alexey Beletsky, Elena Zakharova, Nadezhda Kostrikina, Pavel Sigalevich, Elena Veslopolova, Nikolay Pimenov

**Affiliations:** 1 Winogradsky Institute of Microbiology, Research Center of Biotechnology, Russian Academy of Sciences, 117312 Moscow, Russia; vgorlenko@mail.ru (V.G.); savvichev@mail.ru (A.S.); rusanov_igor@mail.ru (I.R.); vilenta@gmail.com (E.Z.); nadin-kost@yandex.ru (N.K.); pavelsigalevich@mail.ru (P.S.); veslpl@mail.ru (E.V.); npimenov@mail.ru (N.P.); 2 K.G. Skryabin Institute of Bioengineering, Research Center of Biotechnology, Russian Academy of Sciences, 117312 Moscow, Russia; mortu@yandex.ru

**Keywords:** anoxygenic phototrophic bacteria, meromictic karst lakes, lake Kichier, microbial community, biodiversity, *Chlorobium clathratiforme*, *Chlorochromatium magnum*, *Ancalochloris perfilievii*, *Thiopedia rosea*

## Abstract

The rates of oxygenic and anoxygenic photosynthesis, the microorganisms responsible for these processes, and the hydrochemical characteristics of the sulfide-containing karst lakes, Black Kichier and Big Kichier (Mari El Republic), were investigated. In these lakes, a plate of anoxygenic phototrophic bacteria (APB) is formed at the upper boundary of sulfide occurrence in the water. The phototrophic community of the chemocline zone was analyzed using a combination of high-throughput sequencing of the 16S rRNA gene fragments and light and electron microscopic techniques. Green-colored *Chlorobium clathratiforme* were absolutely predominant in both lakes. The minor components included green sulfur bacteria (GSB) *Chlorobium* spp., symbiotic consortia *Chlorochromatium magnum* and *Pelochromatium roseum*, purple sulfur bacteria (PSB) *Chromatium okenii*, and unidentified phylotypes of the family *Chromatiaceae*, as well as members of the *Chloroflexota*: *Chloronema* sp. and *Oscillochloris* sp. Based on the results of the molecular analysis, the taxonomic status of *Ancalochloris perfilievii* and other prosthecate GSB, as well as of the PSB *Thiopedia rosea*, which were visually revealed in the studied freshwater lakes, is discussed.

## 1. Introduction

Meromictic lakes are good research subjects for microbiologists because of the high vertical stability of the water column, which results in a relatively constant stratification of microbial populations. In these basins, a clear vertical heterogeneity is observed in the chemocline, which is a narrow zone containing oxic and anoxic layers separated by a transition layer [1,2]. In the ecosystem formed in the chemocline (redox zone), aerobic and strictly anaerobic microorganisms with diverse types of metabolisms occur together and interact with each other [3]. If light is available, in this zone, anoxygenic phototrophic bacteria (APB) occupying specific layers predominate depending on the illumination intensity and its spectral characteristics, as well as on the oxygen tolerance of the relevant species [4]. The composition of the chemocline microbial communities in meromictic lakes may be compared to the zonal structure of the benthic microbial mats [5]. Both types of microbial communities are considered among the relic ones, which have been predominant at some stages of the Global Ocean evolution [6,7,8]. This adds interest to the in-depth investigation of biodiversity, the mechanisms of interaction, and the final results of the biogeochemical activity of microbial communities in the present-day analogs of ancient ecosystems, including meromictic lakes.

Two main types of meromictic basins exist: those enriched with sulfide, and those enriched with ferrous iron salts [6]. These two substrates may act as electron donors for photosynthesis, so that in these types of basins, sulfide-oxidizing phototrophs and ferrophototrophs develop, respectively [9]. The total mineralization value is another important ecological factor, resulting in the development of freshwater or halophilic microorganisms [10]. Low mineralized karst lakes with sulfide in the monimolimnion constitute a specific type of stratified freshwater basins [1,2]. Lake Kichier, the subject of our study, belongs to this type. While its biogeochemistry has been thoroughly studied, the biodiversity of the APB of the bacterial plate has been described by techniques which are no longer valid.

The accurate planning of research on the diversity of microorganisms is of key importance to ecologists. This certainly applies to APB, which often play crucial parts in the processes occurring in planktonic communities. Pioneering works accumulated an extensive set of data on APB species in the chemocline of various stratified lakes. These data were based on visual assessments of morphology as determined by light or electron microscopy [4]. In our opinion, this primary information is highly important, since it provides the starting point and the vector for a further in-depth study of biodiversity using present-day techniques. New methods for the molecular identification of microorganisms are currently used for the investigation of microbial diversity [11,12]. The application of metagenomic and proteomic techniques provides information, apart from the taxonomic status, also on the potential physiological properties of the taxa revealed in the community, thus supporting suggestions on their activities in such complex microbial communities as bacterial plates in meromictic lakes [13,14,15,16]. Unfortunately, most of the recent molecular ecological works had no relation to the basis of the classical ecological studies; this discrepancy certainly affects their values. Most of the uncultured APB species with unusual morphology described in the literature remain unidentified [9,17].

The Kichier karst lake has long been a subject of hydrochemical and microbiological investigation [18,19,20,21] and may be considered a classical object with the simultaneous presence of almost all known freshwater APB species, including uncultured morphotypes. For these reasons, the lake is promising for an in-depth investigation of the APB biodiversity.

The objectives of this work are to assess the species diversity of phototrophic microorganisms (primarily anoxygenic phototrophic bacteria) in the microbial communities of the chemocline zone of stratified karst lakes. The fundamental novelty of this work lies in the non-standard approach to assessing microbial biodiversity, namely, in the combination of a traditional phenotypic assessment of biodiversity (results of light, fluorescence, and electron microscopy of natural samples) with a careful analysis of phylogenetic trees constructed according to data not only of the dominant OTU values, but also minor OTUs, revealing the presence of minor components of the phototrophic community.

## 2. Materials and Methods

Lake Kichier (Kucheger) (56.07036′ N, 48.34592′ E) is located in the Marii Chodra National Park. It consists of two connected basins, Black Kichier and Big Kichier (Figure S1). The lake was formed due to the dissolution of the Permian limestone layers by ground water. The lake is affected by highly mineralized sulfate waters from deep Paleozoic aquifers, while the upper horizons are influenced by the freshwater surface flow. The total mineralization of deep water is ~2 g L^−1^. The differences in density hinder the mixing of the water column and favor stratification [17]. Due to considerable sulfate concentration, anaerobiosis caused by sulfide accumulation in the water and bottom sediments is established, resulting in the formation of a sulfide-dependent community. Black Kichier is relatively small (4 ha), with a maximal depth of ~10 m. It is rounded in shape, shielded at all sides from wind mixing, and is connected with Big Kichier by a narrow girt. The average depth of Big Kichier is 5 m, with two craters (13 and 7 m). In Big Kichier, unlike the meromictic Black Kichier, partially impaired stratification of the water column was observed during the seasonal mixing. This indicated that Big Kichier was a dimictic basin [17].

### 2.1. Sampling

Sampling for hydrochemical and biogeochemical investigation of the water column and for the determination of microbial abundance and composition in the chemocline zone was carried out in late July 2022 at the sites with the greatest depth during the sampling time (8 m for Black Kichier and 10 m for Big Kichier (Figure S1).

Water samples were collected with a Ruttner bathometer at the minimal intervals of 0.5 m. Illumination at different horizons of the water column was determined with an AR813A Luxmeter (Smart Sensor, China), which was modified for underwater operation. Redox potential (Eh and mV), pH, and temperature were determined using a WTW 3320 SET2 portable potentiometer with temperature compensation (Weilheim, Germany). Sulfide was determined by iodometric titration. Oxygen was determined using the Winkler method. Carbonate carbon was determined by acidometric titration with methyl orange and phenolphthalein as pH indicators.

### 2.2. Microscopy and Pigment Composition

To determine the total microbial abundance (TMA) and microbial morphological diversity, water samples were fixed with glutaraldehyde at 2% final concentration in the sample. Fixed samples (15 mL) were filtered through 0.2 µm black polycarbonate membranes (Millipore). The filters were stained with Acridine orange. The preparations were examined at ×1000 under an Axio Imager D1 epifluorescence microscope (Carl Zeiss, Jena, Germany) equipped with an Image Scope Color (M) visualization system. Biomass was calculated using the data on the volume of microbial cells and assuming 1.0 mg mm^−3^ as the density of wet biomass. Specific biomass of microbial cells (B) is therefore presented in micrograms per liter (µg L^−1^). During microscopic examination of the stained preparations, single cells and aggregated cells were enumerated separately. A group of cells with a common outline, in which visual enumeration of the individual cells was difficult or impossible, was considered an aggregate.

Bacterial morphology was studied under an Olympus BX 41 light microscope (Tokyo, Japan) with a ×100 phase objective. The preparations for electron microscopy were prepared as follows: To obtain negatively stained whole cell preparations, bacterial suspensions were applied to formvar-coated copper grids and stained with 0.2% aqueous uranyl acetate. For ultrathin sections, the material was processed according to Kellenberger [22], dehydrated, and embedded in Epon. The sections were placed on formvar-coated copper grids and contrasted by lead citrate [23]. The preparations were examined under a JEM-100B (JEOL, Tokyo, Japan) at 80 or 90 kV.

Pigment composition was determined by absorption spectra obtained on an SF-56 spectrophotometer (LOMO, St. Petersburg, Russia) within the wavelength range of 350–1000 nm. Bacterial suspensions were prepared in 50% glycerol. Quantitative determination of the chlorophylls was carried out using ethanol extracts from the cells concentrated on 47 mm GF/F glass fiber filters from 0.5–1.5 L of lake water. Spectral characteristics of extracted cell pigments were studied, and the concentration of bacteriochlorophylls *d* + *e* was calculated according to [24].

### 2.3. Radiotracer Experiments

The rates of photosynthesis and microbial CO_2_ assimilation were determined by the radiocarbon method with NaH^14^CO_3_, according to the standard procedure [25]. Water for determination of the rates of photosynthesis by the phytoplankton and of dark (microbial) carbon assimilation (DCA) was collected with a bathometer and dispensed into 120 mL vials through a silicon rubber tube, avoiding turbulence, with double flushing and 50% overflow. The vials were sealed (avoiding air bubbles) with rubber stoppers fixed with perforated aluminum caps. The samples were dispensed in the dark. At each horizon, two transparent vials and two dark ones (covered with aluminum foil) were used for rates of photosynthesis and DCA determination, respectively. The dark vials were the controls for the transparent ones. For the DCA control, a dark vial was supplemented with 1 mL 0.05 N HCl. Sterile NaH^14^CO_3_ solution (100 µL, specific activity 2.04 GBk mmol^−1^, 5 µCi/sample) was injected in the dark into all vials. DCMU (3-(3,4)dichlorophenyl-1,1-dimethylurea) at the final concentration of 10^−7^ mM was used to inhibit oxygenic photosynthesis. The shared darkening sheaths were then removed, and the vials were attached to the horizontal beams attached to the incubation line at the sites corresponding to the sampling horizons. Immersed vials were incubated in situ for half of the daylight hours at the buoy station located at the sampling site. After exposure, the samples were fixed with 1 mL of 0.05 N HCl. Determination of primary production and DCA was carried out as described previously [26]. The total photosynthetic production was calculated as the difference between the values for the dark and transparent values. Production of oxygenic photosynthesis was calculated as the difference between the total and anoxygenic photosynthesis in transparent vials with DCMU. Production of microbial CO_2_ assimilation was calculated as the difference between the values for the dark and control vials.

### 2.4. Isolation of Metagenomic DNA, PCR Amplification, and High-Throughput Sequencing of the 16S rRNA Gene Fragments

Immediately after collection, water samples (200 mL) were filtered through 0.22 µm membranes. The filters were homogenized by grinding with liquid nitrogen, and the metagenomic DNA preparation was obtained using the Power Soil DNA Isolation Kit (MO BIO Laboratories, Carlsbad, CA, USA). The composition of the prokaryotic community was determined by analyzing the sequences of the V3–V4 variable region of the 16S rRNA gene, amplified by PCR using the primers PRK341F (5′-CCTACGGGRBGCASCAG-3′) and PRK806R (5′-GGACTACYVGGGTATCTAAT-3′). The obtained PCR fragments were used to construct the library using the Nextera XT DNA Library PrepKit (Illumina, San Diego, CA, USA) according to the manufacturer’s protocols. The libraries were sequenced on MiSeq (Illumina, San Diego, CA, USA) using MiSeq Reagent Kit V3 (in the paired reads format 2 × 300 nt). The obtained reads were combined using the FLASH v 1.2.11 software package [27].

A total of 48,153 sequences of the 16S rRNA gene fragments per sample were obtained. Each set of sequences was clustered into operational taxonomic units (OTUs) at 97% identity; the chimeras were removed with Usearch [28]. A 16S phylogenetic tree was inferred using FastTree v2.1.11 from the alignment constructed by MAFFT v7.055b. Default parameters were used for both programs, and local support values were computed with the Shimodaira–Hasegawa test. Taxonomic identification of the OTUs was carried out with SINA using the SILVA database with default parameters [29].

## 3. Results

### 3.1. Physicochemical Conditions in the Black and Big Kichier Lakes

**Figure 1:**
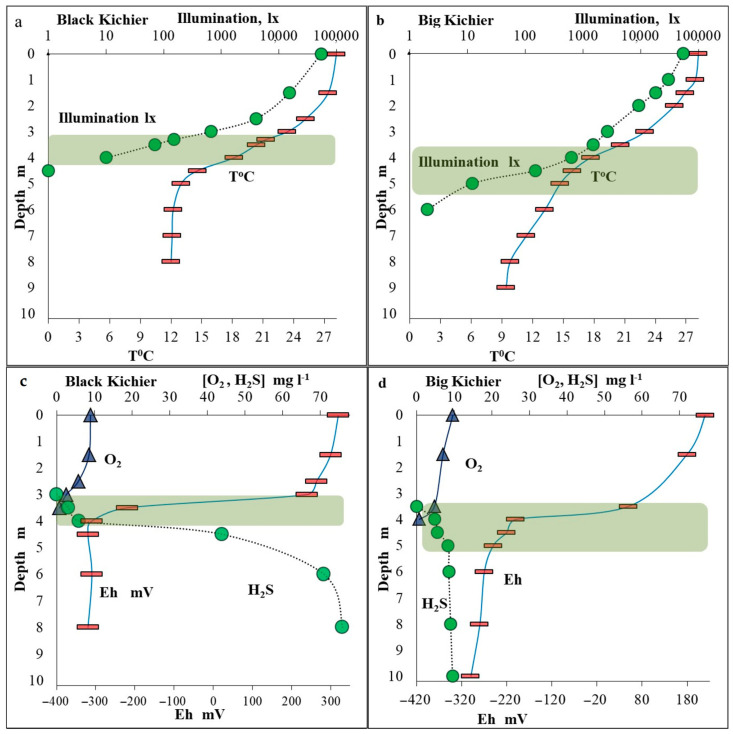
Temperature and illumination in the water column of the Black Kichier (**a**) and Big Kichier (**b**) lakes. Concentrations of oxygen and sulfide (mg L^−1^) and redox potential (mV) in the water column of the Black Kichier (**c**) and Big Kichier (**d**) lakes. Location of the bacterial plate is indicated by green shading.

In both lakes, the hydrochemical characteristics and the distribution of microorganisms revealed pronounced zonality of the water column. The weather at the time of sampling was hot, and the temperature of the surface water layer was 27.5 °C (Figure 1). The mixed layer thickness was up to 2.0 m. In Black Kichier, a pronounced thermocline was located at the depth of 3.0–4.0 m. In Big Kichier, the thermocline, located at 3.5–4.5 m, was less pronounced. At the time of sampling, the transparency of the water column was low in both lakes. Illumination measurements revealed that the most optically dense water layer, where the bacterial plate developed, was located at 3.5–4.0 and 4.0–5.0 m in the Black and Big Kichier lakes, respectively.

The oxygen concentration at the surface of both basins was saturating (9–9.5 mg L^−1^) (Figure 1). Below 2 m, the oxygen saturation began to decrease, reaching zero at 4.0 and 4.2 m in Black and Big Kichier, respectively. At these depths, the sulfide smell was pronounced in the water samples. The sulfide concentrations increased sharply with depth, reaching 76 and 9.5 mg L^−1^ in the near-bottom water layers of the Black and Big Kichier lakes, respectively. The location of the chemocline zone in the water column was most clearly visible by a decrease in Eh values (redox potential). The Eh drop was observed in the Black and Big Kichier lakes at the depths of 3.5 and 4.0 m, respectively.

### 3.2. Chlorophylls

The spectral characteristics of the pigments of the cells collected from the chemocline zone of the Black and Big Kichier lakes were almost identical. The absorption spectra of the cells from a chemocline water sample (3.5 m) of Black Kichier and of the ethanol extract from the cells are shown in Figure S2. For whole cells, the main long-wavelength maximum was observed at 725 nm, indicating the predominance of bacteriochlorophylls *d+e*-containing bacteria [30]. Chlorophyll *a*-containing oxygenic phototrophs were responsible for the peak at 665 nm. The presence of trace amounts of bacteriochlorophyll *a* was indicated by the shoulder at ~800 nm. Judging by the height of the long-wavelength peaks, the content of bacteriochlorophylls in the samples was about twice that of chlorophyll *a*. The ethanol extracts of chlorophylls revealed a predominance of bacteria containing BChl *d* (maximum at 657 nm) in the chemocline of both lakes.

### 3.3. Total Microbial Abundance (MA)

**Figure 2:**
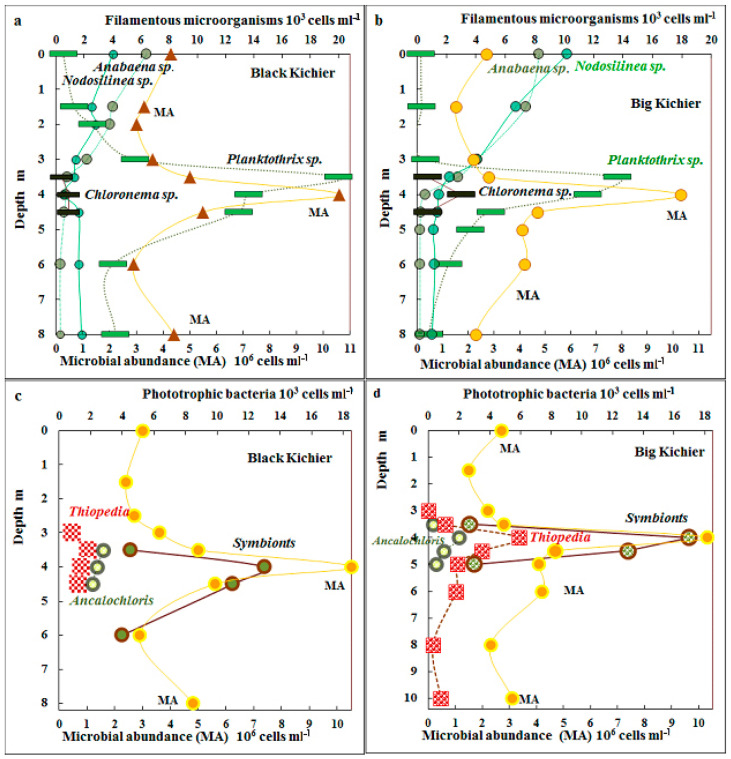
Total microbial abundance (MA), 10^6^ cells mL^−1^ (lower scale) and numbers of filamentous microorganisms (*Anabaena sp.*, *Nodosilinea sp.*, *Chloronema sp.*, and *Planktothrix sp.*) (10^3^ cells mL^−1^, upper scale) in the water columns of Black Kichier (**a**) and Big Kichier (**b**). MA, 10^6^ cells mL^−1^ (lower scale) and abundance of *Thiopedia*, *Ancalochloris*, and symbionts, 10^3^ cells mL^−1^ (upper scale) in the water columns of the Black Kichier (**c**) and Big Kichier (**d**) lakes.

The MA peaked in the chemocline zone, where it was as high as 10.5 × 10^6^ and 10.2 × 10^6^ cells mL^−1^ in Black Kichier and Big Kichier, respectively (Figure 2). This maximum coincided with the maximum for bacteriochlorophylls *d* + *e*, the pigments of GSB that are predominant in this zone.

### 3.4. Cyanobacteria

The distribution of morphologically discernible species of cyanobacteria and APB in the water column is shown in Figure 2. Unicellular cyanobacteria morphologically similar to *Sinechococcus* were predominant in the mixolimnion (from 300 to 470 × 10^3^ cells mL^−1^). Filamentous cyanobacteria of the genus *Nodosilinea* sp. (=*Leptolyngbya* sp.) were also present in this zone, although their abundance did not exceed 6 × 10^3^ cells mL^−1^. The highest cyanobacterial numbers were observed in the chemocline zone, where both unicellular *Sinechococcus* (450–670 × 10^3^ cells mL^−1^) and filamentous *Planktothrix* sp. were present. Their numbers were as high as 20 × 10^3^ cells mL^−1^ in Black Kichier and 14 × 10^3^ cells mL^−1^ in Big Kichier. An unusual location of filamentous cyanobacteria in the chemocline of Lake Kichier was observed by Gorbunov and Umanskaya in June 2009 [17].

### 3.5. Anoxygenic Phototrophic Bacteria

Anoxygenic phototrophic bacteria were mainly represented by GSB. The cells of the dominant species *Chlorobium* (=*Pelodictyon*) *clathratiforme* could not be counted due to the disintegration of microcolonies characteristic of the species during filtration. Microscopy revealed the symbiotic *Chlorochromatium* and *Pelochromatium* species (12–16 × 10^3^ cells mL^−1^), as well as *Ancalochloris* sp. (2–3 × 10^3^ cells mL^−1^). The abundance of the purple bacteria morphologically identified as “*Thiopedia rosea*” was as high as 2–4.5 × 10^3^ cells mL^−1^, and that of the filamentous *Chloronema*–like [31] was up to 2–4 × 10^3^ cells mL^−1^ (Figure 2).

### 3.6. Production Processes

**Figure 3:**
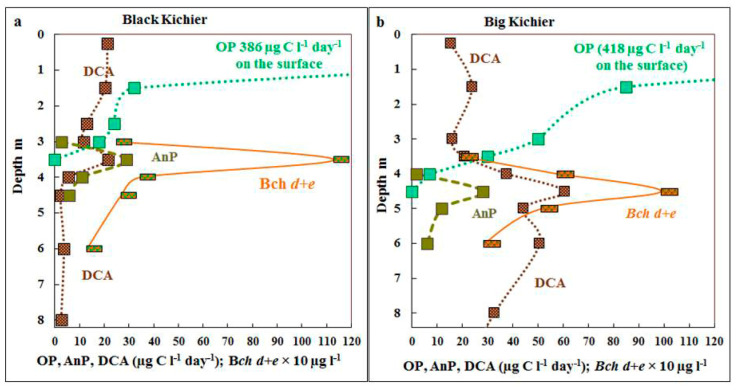
Rates of oxygenic (OP) and anoxygenic (AnP) photosynthesis and dark CO_2_ assimilation (DCA), µg C L^−1^ day^−1^ as well as bacteriochlorophyll *d + e* (Bch *d + e*, 10 µg L^−1^) in the water column of Black Kichier (**a**) and Big Kichier (**b**).

The highest rate of oxygenic photosynthesis was observed in the surface water layer, where it reached 386 µg C L^−1^ day^−1^ in Black Kichier and 418 µg C L^−1^ day^−1^ in Big Kichier (Figure 3). Its rate decreased rapidly with depth, and at 1.5 m, it was 32 and 85 µg C L^−1^ day^−1^ for Black and Big Kichier, respectively. No oxygenic photosynthesis was detected at 3.5 m in Black Kichier and at 4.5 m in Big Kichier. The calculated integral production of oxygenic photosynthesis was 350 mg C m^−2^ day^−1^ in Black Kichier and 630 mg C m^−2^ day^−1^ in Big Kichier. The differences in integral photosynthetic production were probably due to the earlier onset of the short bloom of planktonic algae in Black Kichier, which has already passed by the time of sampling.

The maximum of anoxygenic photosynthesis was observed at the depth where oxygen disappeared, and the rate of the process was relatively low (25–30 µg C L^−1^ day^−1^), which resulted either from extremely low illumination in this zone or from the probably toxic products of oxygenic phototrophs, since their bloom ended close to the sampling time. The rate of bacterial photosynthesis was 10–15 times lower than that of the oxygenic one, and in late July, its contribution to the total production was not high (<8% in Black Kichier and <6% in Big Kichier).

### 3.7. Biodiversity of Bacteria in the Chemocline of Black Kichier and Big Kichier

**Figure 4:**
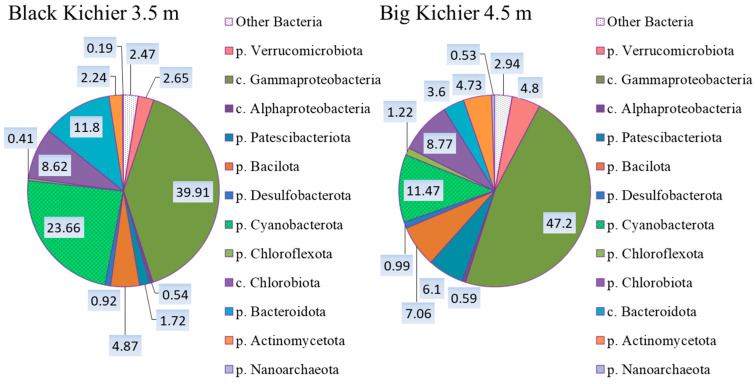
The composition (%) of bacterial plate chemocline microbial communities in Black Kichier and Big Kichier according to the results of high-throughput sequencing of the 16S rRNA gene fragments.

According to the results of the high-throughput sequencing of the 16S rRNA gene fragments, the compositions of the microbial communities of the chemocline bacterial plate in two lakes were approximately the same (Figure 4). Members of Gammaproteobacteria predominated (40–47%). *Cyanobacterota* were the second most abundant group (11–23%). The shares of *Chlorobiota* in both lakes slightly exceeded 8%, while the relative abundance of *Chloroflexota* did not exceed 1.2%.

**Figure 5:**
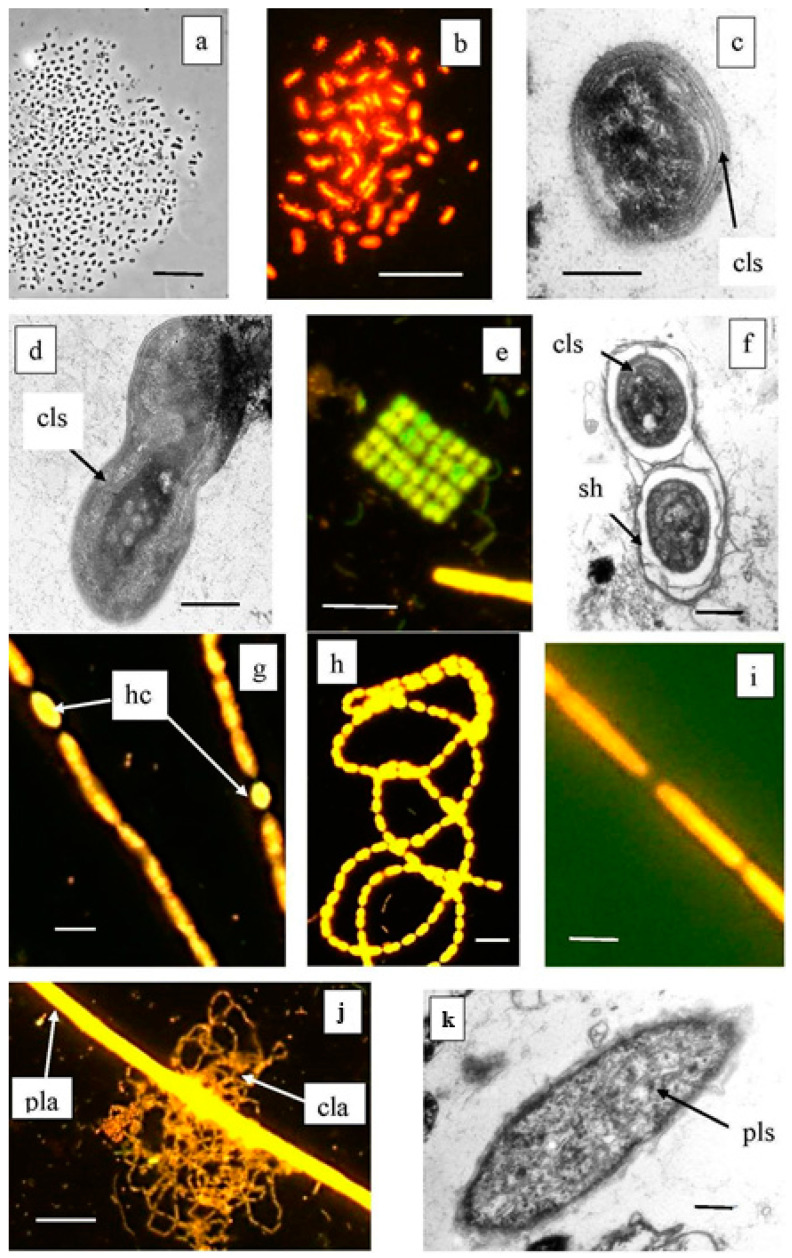
Cyanobacteria from Lake Black Kichier. Note: (**a**) Phase contrast; (**b**,**e**,**g**–**i,j**) fluorescent microscope, (**c**,**d**,**f**,**k**) TEM, ultrathin section; (**a**–**d**) morphotypes of *Synechococcus—Cyanobium*; (**e**,**f**) *Merismopedia* sp.; (**g**,**h**) *Anabaena* sp. and *Leptolyngbia* sp.; (**j**) thread of *Planktothrix* sp. along with a reticulate colony of *C. clathratiforme*. (**k**) Ultrathin section of *Planktothrix* sp., lamellar photosynthetic structures are visible; cls—ring photosynthetic lamellae; hs—heterocysts; sh—mucous cover; pla—*Planctothrix* sp.; cla—*C. clathratiforme*; pls—photosynthetic lamellar structures. Magnification (bar): (**a**,**b**,**e**,**j**) 10 µ; (**g**,**h**) 5 µ; (**i**) 2 µ; (**c**,**d**,**f**,**k**) 1 µ.

An in-depth analysis made it possible to determine the dominant and minor species of phototrophic bacteria in the bacterial plates of both lakes. Cyanobacteria were found to predominate in the redox zones of both lakes, while the abundance of GSB was 3–4 times lower compared to the oxygenic prokaryotes. The microscopy revealed the following cyanobacterial morphotypes: filamentous *Planktothrix*, *Leptolingbya*, *Anabaena*, unicellular *Sinechococcus*-like morphotypes, and *Merismopedia* (Figure 5).

#### 3.7.1. Cyanobacterota

**Figure 6:**
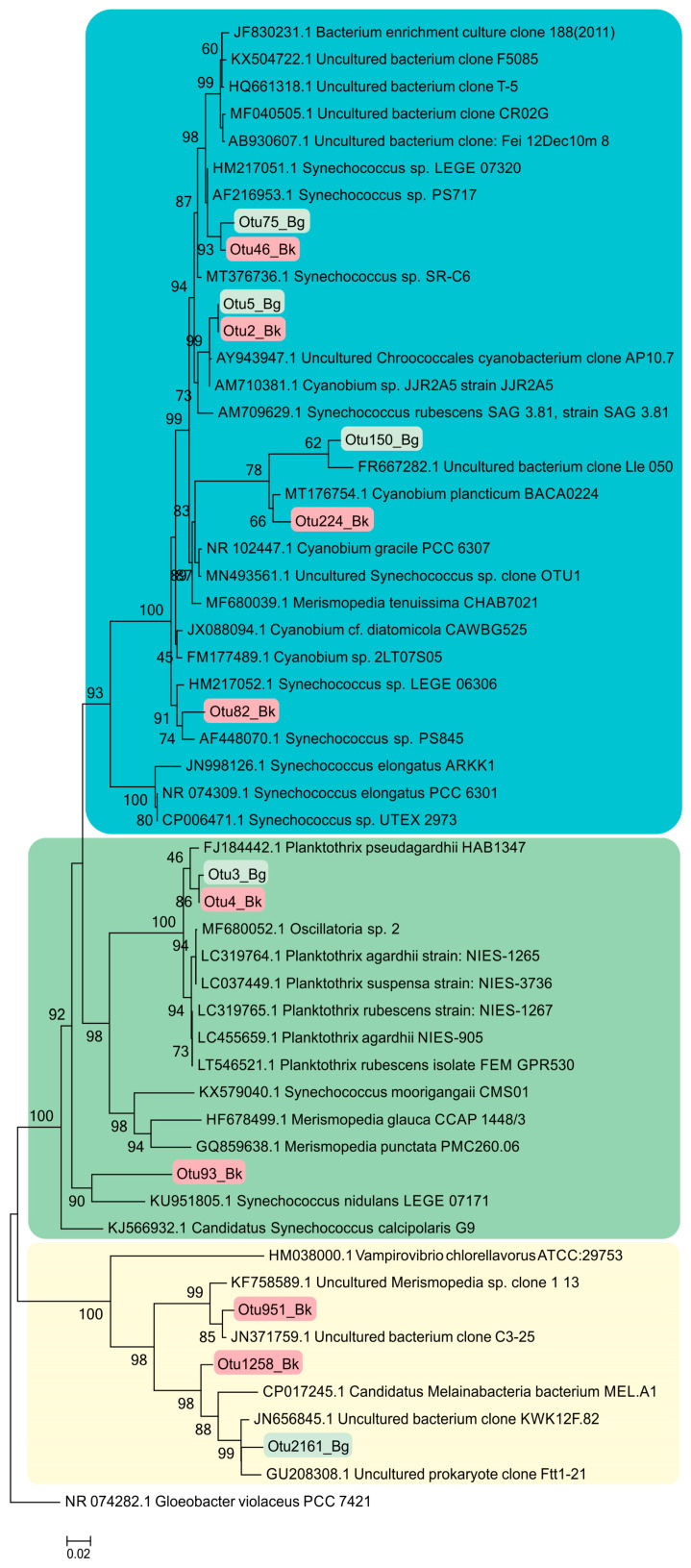
Phylogenetic position of the main members of the *Cyanobacterota* phylum according to the results of high-throughput sequencing of the 16S rRNA gene fragments in the Black Kichier (Bk) and Big Kichier (Bg) lakes.

**Figure 7:**
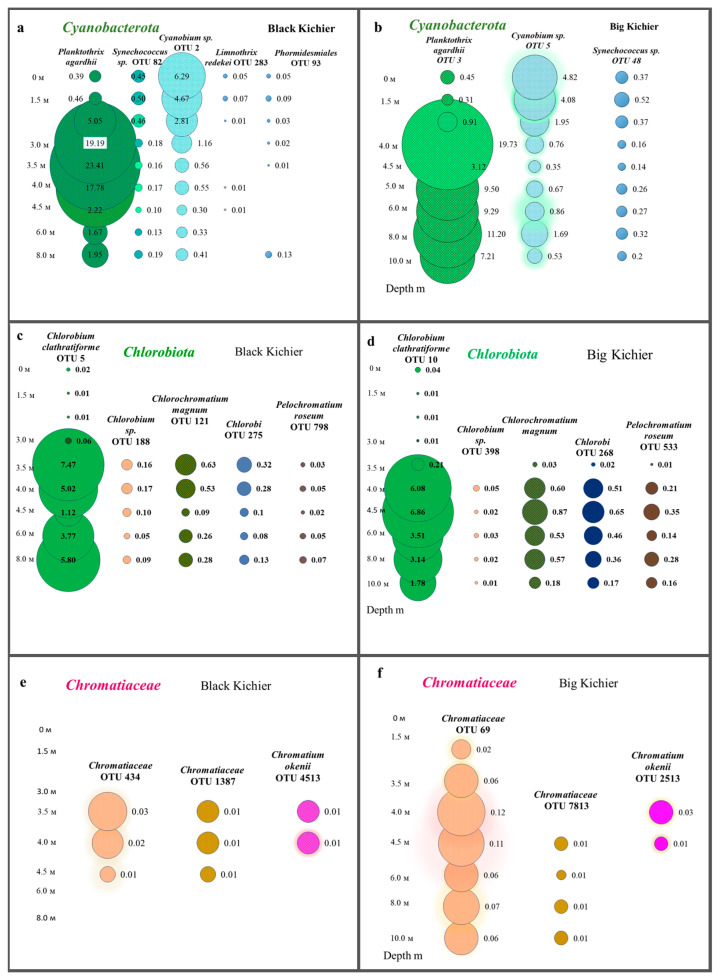
Distribution of cyanobacteria (**a**,**b**), Chlorobia (**c**,**d**), and Chromatiaceae (**e**,**f**) (OTU, %) in the water column of the Black and Big Kichier lakes.

Molecular techniques were used to specify the taxonomic position of cyanobacteria inhabiting the Black (Bk) and Big Kichier (Bg) lakes (Figure 6). Three clusters were visible on the phylogenetic tree. The upper one comprised a diverse group of unicellular cyanobacteria of the genera *Sinechococcus* and *Cyanobium* [32]. They occurred in the oxic zone of the epilimnion, and their development was probably due to the second wave of blooming. *Planktothrix agardii* (OTU 3, 98.87% similarity to *Planktothrix agardhii* NIES-905) was predominant in both Kichier lakes. Members of this genus are known to produce microcystin and cause cyanobacterial blooms in diverse regions, especially in the temperate zone of the Northern Hemisphere [33]. This species was a component of the second cluster, together with other filamentous cyanobacteria. The relative abundance of *Planktothrix agardii* in Lake Kichier was as high as 23.41% of the total number of the nucleotide sequences; its maximal abundance was found at the upper boundary of the chemocline. Such distribution of filamentous cyanobacteria has been previously reported for Lake Kichier [17]. The accumulation of this species in the chemocline zone may be explained by the precipitation of cyanobacterial cells after the massive bloom. *Merismopedia* sp. was a minor species concentrated in the chemocline. The cyanobacteria of this group were components of the third cluster, which also included *Vampirovibrio chlorellavorus*. The distribution of predominant cyanobacterial phylotypes in the water column of the studied lakes is shown in Figure 7.

#### 3.7.2. Chlorobia

**Figure 8:**
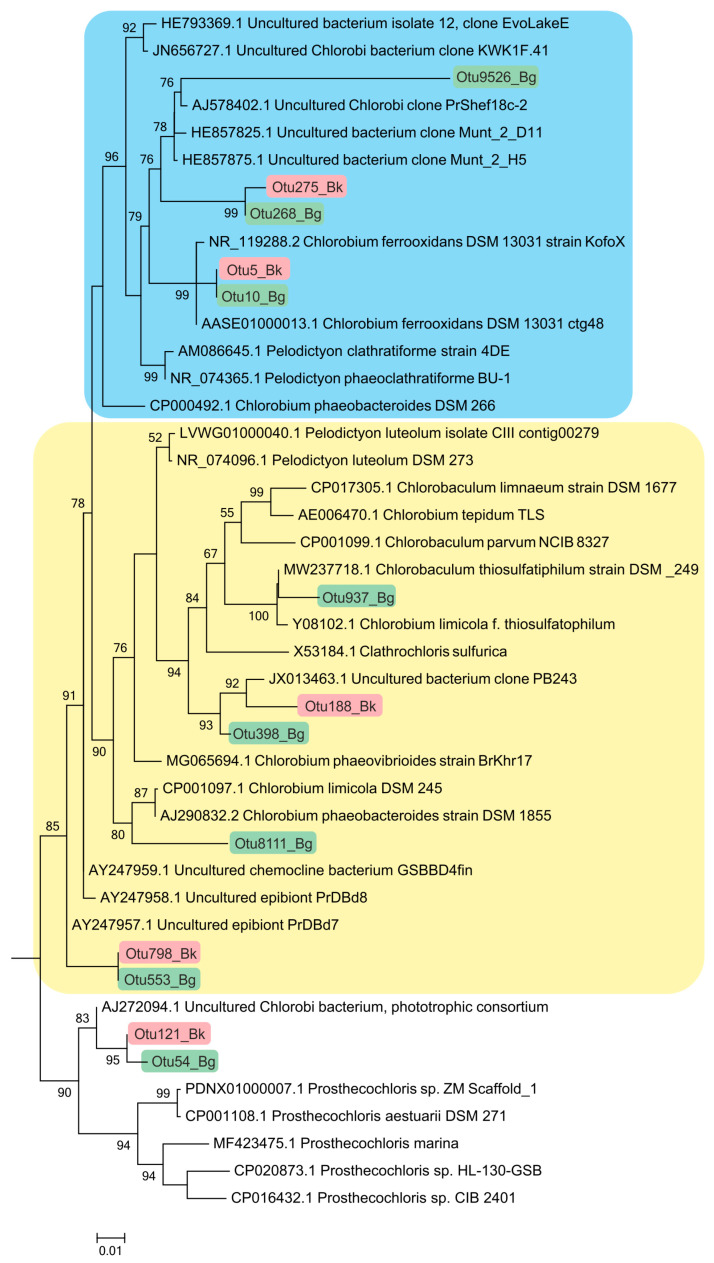
Phylogenetic positions of the main members of the class *Chlorobia* according to the results of high-throughput sequencing of the 16S rRNA gene fragments in the Black Kichier (Bk) and Big Kichier (Bg) lakes.

**Figure 9:**
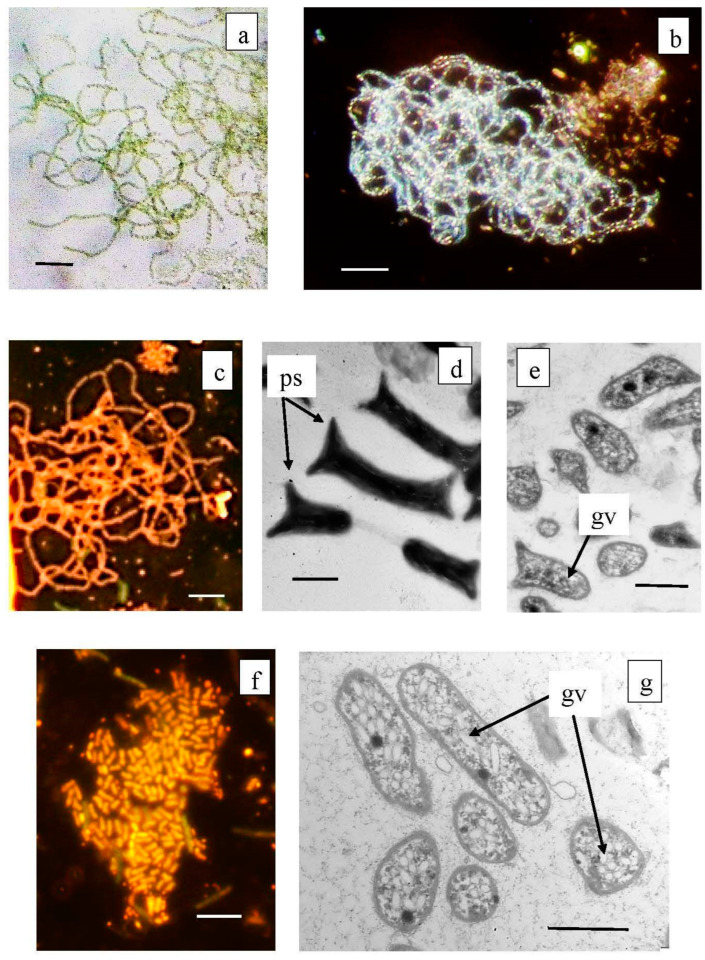
Microphotographs *of Chlorobium clathratiforme* dominating among anoxygenic phototrophs in the Black Kichier lake (**a**–**e**), and minor species of *Chlorobium luteolum* (**f**,**g**). Note: Light microscope ((**a**) bright field; (**b**) dark field; (**c,f**) fluorescent microscope); TEM: (**d**) total ells; (**c**,**g**) ultra thine section; ps—prosteke; gv—gas vesicles. Magnification (bar): (**a**–**c**,**f**) 5 µ; (**d**,**e**,**g**) 1 µ.

Members of the class *Chlorobia* were predominant in the bacterial plate among the anoxygenic phototrophic bacteria (Figure 7c). The high-throughput sequencing of the 16S rRNA gene fragments revealed three phylogenetic clusters (Figure 8). The first one probably comprised the GSB with cell prosthecae. Bacteria of OTU 5_Bk and OTU 10_Bg with 99.25% similarity to *Chlorobium clathratiforme* (complete sequence of *Pelodictyon phaeoclathratiforme* BU-1) and *Chlorobium ferrooxidans* (DSM 13031 strain KofoX) predominated in the community. Since *Chlorobium ferrooxidans* is common in low-sulfide lakes enriched in ferrous iron, it can be argued that the green-colored *Chlorobium clathratiforme*, constantly noted by other researchers in this lake, is present and dominant in the Kichier lakes (Figure 9). This GSB was often found in sulfide-enriched freshwater-stratified lakes as a dominant and minor APB species [26,34,35,36].

**Figure 10:**
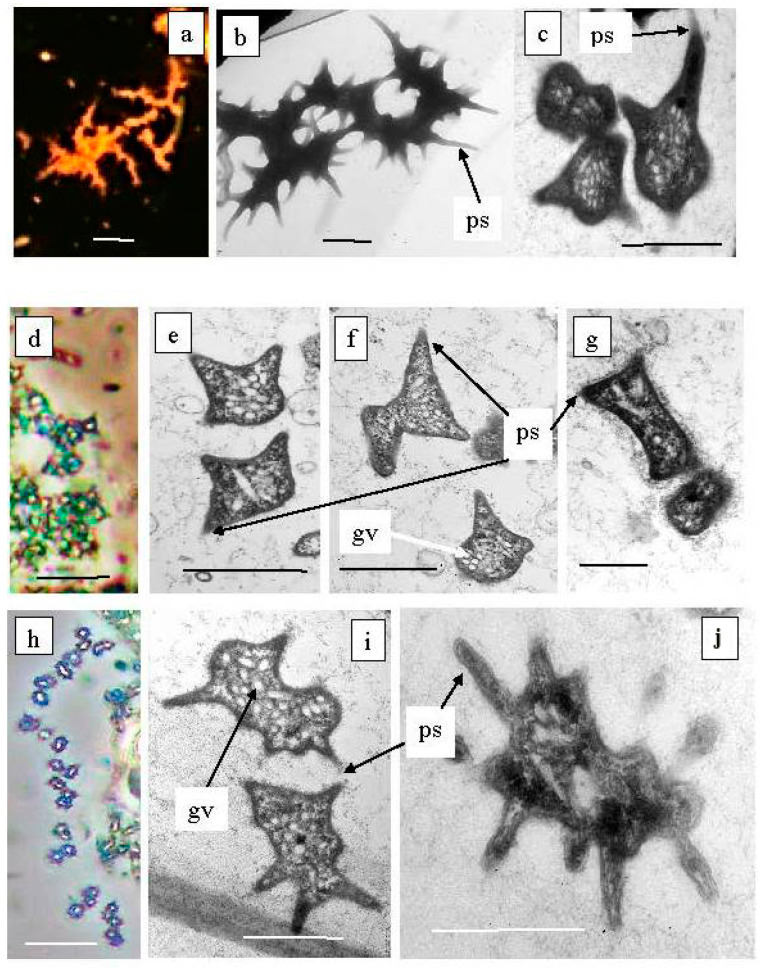
Green sulfur bacteria of unusual morphology living in the Black Kichier lake. Note: (**a**) fluorescent microscope; (**d**,**g**) light microscope phase contrast. Electron microscope: (**b**) total cells; (**c**,**e**–**g**,**i**,**j**) ultrathin sections; ps—prosteke; gv—gas vesicles; *Ancalochloris perfilievii* (**a**–**c**) forming long streaks; *Ancalochloris perfilievii* form 1, angular irregular shape (**d**–**f**); *Ancalochloris* sp. form 2, *Prosthecochloris* of similar morphology (**h**–**j**); *Chlorobium clathratiforme* (**g**). Magnification (bar): (**a**,**d**,**h**) 5 µ; (**b**,**c**,**e**–**g**,**i**,**j**) 1 µ.

The abundance values of *Chl. clathratiforme* in the chemocline of Black Kichier (OTU 5_Bk) and Big Kichier (OTU 10_Bg) were 7.47 and 6.86% of all prokaryotic microorganisms, respectively. The unidentified GSB OTU 275_Bk, OTU 268_Bg, and OTU 9526_Bg, distantly related to the dominant (96.24% similarity with *Chl. clathratiforme*), belonged to the same cluster. These GSB with an uncertain taxonomic position probably belonged to one of the morphotypes similar to *Ancalochloris*, which were observed in the lakes (Figure 10).

*Ancalochloris perfilievii* was originally found in 1967 in both Black and Big Kichier. This species was subsequently studied in an enrichment culture and validated [37]. In Lake Kichier, *Ancalochloris perfilievii* has been constantly present as a subdominant during the past 55 years of observation [17,21]. These bacteria have prosthecae—long cellular outgrowths with a wide base (Figure 10a–c). The second morphological form includes bacteria with angular cells (Figure 10d–g). Bacteria of the *Ancalochloris* sp. (third morphotype, Figure 10h,i,k) have cells with short thin prosthecae, morphologically reminiscent of the marine species *Prostecochloris aestuarii.*

The presence of GSB with various types of prosthecae has been repeatedly noted for stratified freshwater lakes with low sulfide contents [38,39,40]. Skuia named the *Ancalochromis perfilievii*-like morphotype *Clathrohloris hypolimnica* [41]. GSB from Lake Michigan that had short, thin projections were named *Prosthecochromis* sp. [42]. It should be noted that the GSB morphotype with angular cells has not previously been observed in freshwater stratified lakes.

#### 3.7.3. Symbiotic Forms of GSB

The second phylogenetic cluster comprises GSB without prosthecae and not participating in symbiotic consortia. Among these, *Chlorobaculum thiosulfidophilum* was reliably identified in Big Kichier (OTU 937, 98.5% similarity). It should be noted that *Chlorobium luteolum* (=*Pelodictyon aggregatum*) were usually predominant in Lake Kichier when cyanobacterial blooms did not occur [17] (Figure 9f,g). This species was found to be dominant in the chemocline of some stratified freshwater lakes located in different geographic zones. In earlier works, this species was termed “*Clathrochloris sulfurica*” [37,38,40,41]. Microorganisms with the nucleotide sequence OTU188_Bk and OTU398_Bg and OTU8111_Bg had a low similarity (95.5–96.64%) with *Chlorobium luteolum* and *Chlorobium limicola* and belonged to unidentified GSB bacteria.

**Figure 11:**
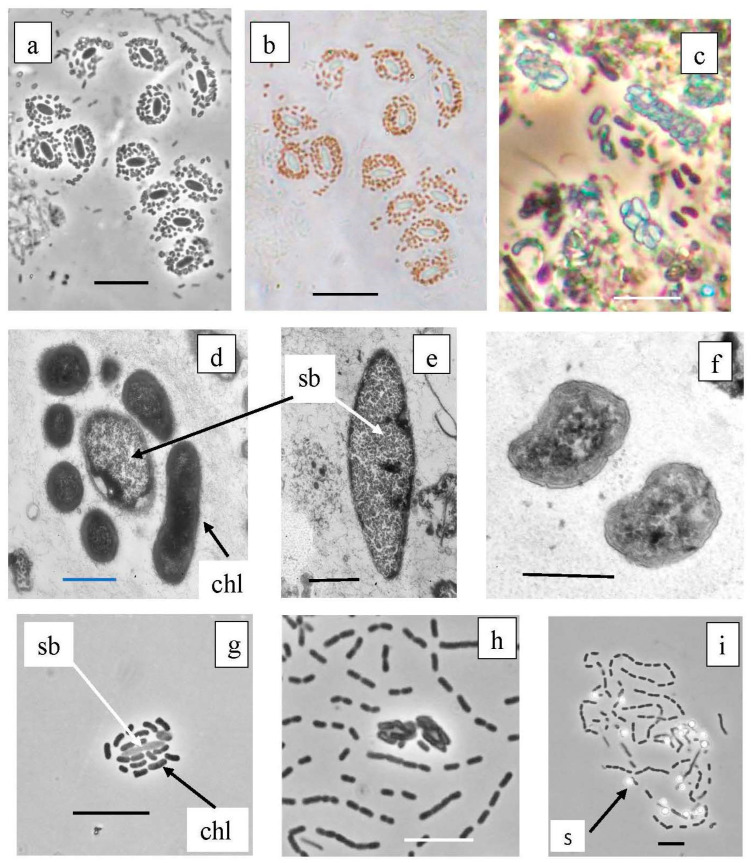
Symbiotic consortia from the chemocline of Black Kichier lake. Note: (**a**,**c**,**g**,**h**) light microscope, phase contrast; (**b**) light microscope, bright field; (**d**–**f**) TEM, ultrathin sections. Magnification (bar): (**a**–**c**,**g**,**h**,**i**) 5 µ; (**d**–**f**) 0.5 µ; (**a**,**b**) *Pelochromatium roseum*, (**c**) *Chlorochromatium magnum*, (**d**) unidentified consortium; (**f**) *Chlorobium* sp., (**g**,**h**) consortium GSB from enrichment cultures. (**i**) Free living GSB form chains of cells, oxidize sulfide to sulfur deposition; sb—*Simbiobacter* sp.; chl—*Chlorobium* sp.; s—sulfur.

Symbiotic forms of GSB were numerous in the plankton of the chemocline of both lakes, Big and Black Kichier (Figure 11, Table 1). The third cluster comprised the green and brown symbiotic morphotypes, as well as environmental clones. Predominant among the symbionts were “*Chlorochromatium magnum*” (99.6% similarity with *Chlorochromatium magnum*), from the chemocline of Lake Dagow, (OTU553 Bg, OTU121 Bk), and to a lesser degree, *Pelochromatium roseum* (98.8–99.2% identity, uncultured Chlorobiota “Pelochromatium roseum” from the chemocline of Lake Schleinsee) (BK 2677 OTU553, BLK 2303 OTU798). The occurrence of “*Chlorochromatium aggregans*” and “*Cylindrogloea bacterifera*” has been previously reported for Lake Kichier. The OTU9526 probably belonged to the GSB of one of these types of symbionts (94.2% identity) [42,43,44,45,46,47].

**Figure 12:**
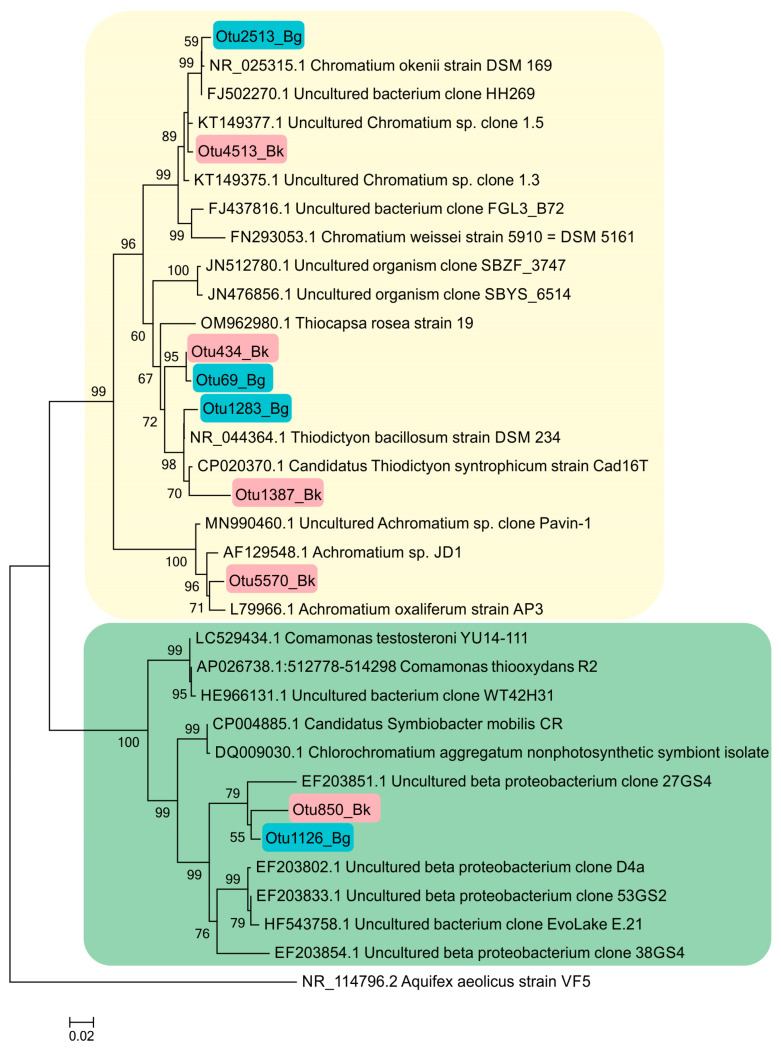
Phylogenetic position of the main representatives of the phylum *Pseudomonadota* according to the results of high-throughput sequencing of the 16S rRNA gene fragments in the Black Kichier (Bk) and Big Kichier (Bg) lakes.

The phylogenetic tree presented in Figure 12 shows the phylogenetic position of *Betaproteobacteria* (*Comamonadacea*), to which the heterotrophic bacteria in the GSB symbiotic consortia belong. The detection of OTU 850_Bk and OTU 1126_Bg indicates the presence bacteria close to *Simbiobacter* sp.

#### 3.7.4. Purple Sulfur Bacteria

**Figure 13:**
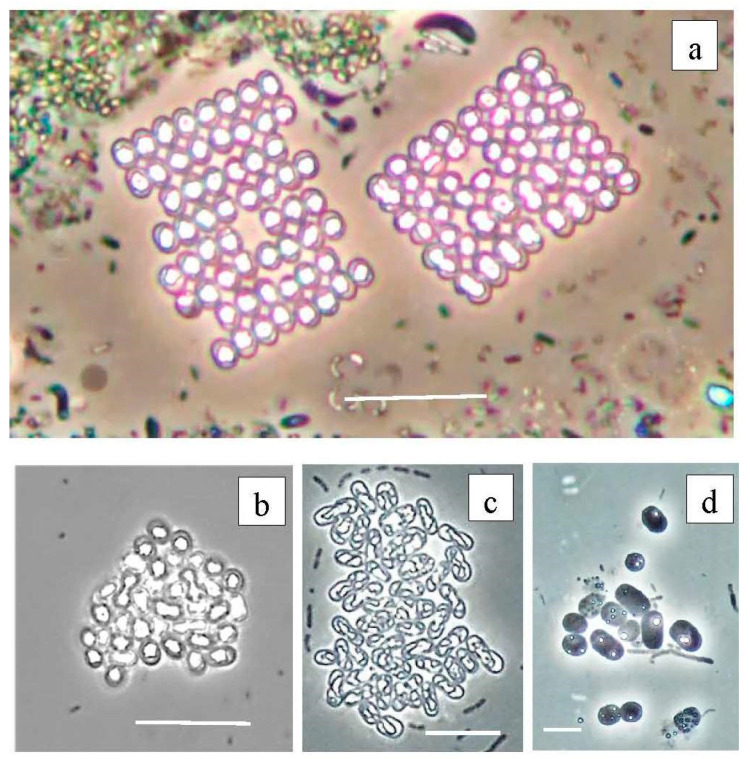
Purple bacteria of the Black Kichier lake. Note: light microscope: (**a**) bright field, (**b**–**d**) phase contrast; (**a**) natural sample from chemocline zone, (**b**–**d**) enrichment culture. (**a**) *Thiopedia rosea*, (**b**) *Thiocapsa rosea*, (**c**) *Thiodictyon elegans*, (**d**) *Chromatium* sp. Magnification (bar): (**a**,**b**) 10 µ; (**c**) 5 µ; (**d**) 2 µ.

According to the results of high-throughput sequencing, the relative abundance of PSB in Lake Black Kichier did not exceed 1% of APB and ~0.04% of all identified bacteria. *Chromatium okenii* was a reliably identified OTU (99.25% similarity to the type species). In Lake Big Kichier, apart from *Chromatium okenii* (OTU 2513_Bg-99.25%), PSB related to *Thiodictyon bacillosum* were also revealed (OTU 1283_Bg-98.87%). These species have been repeatedly identified in freshwater meromictic and dimictic lakes [4,48,49,50]. The 16S rRNA gene fragments OTU 69_Bg and OTU 434_Bk had low similarity (95.13–96.4%) with *Thiodictyon bacillosum*. The exact identification of these PSB proved impossible, but it can be assumed that they belong to *Thiopedia* sp. [51] Among the purple sulfur bacteria of the Black and Big Kichier lakes, *Thiopedia rosea*, with its elegant flat microcolonies, usually consisting of 16 spherical cells with gas vacuoles, was morphologically conspicuous (Figure 13). We also note that during the microscopy of natural samples and in an enrichment culture, PSB of the *Thiocapsa* morphotype were observed (Figure 13b,d).

#### 3.7.5. Planktonic Choroflexaceae Species

**Figure 14:**
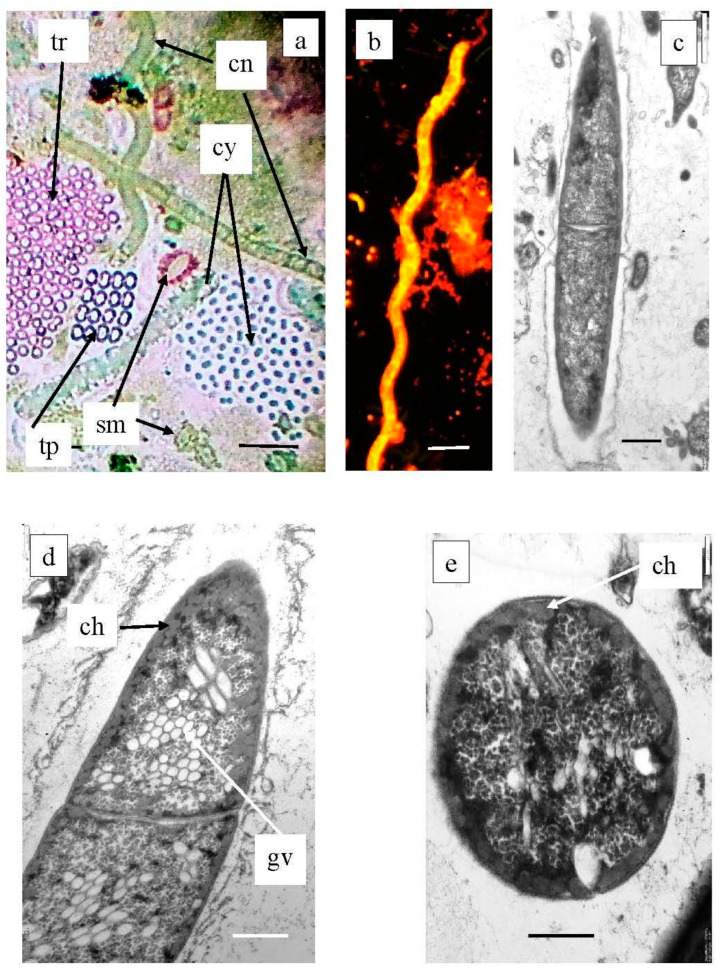
Plankton species of *Chloroflexota* in Black Kichier lake. Note: (**a**) Light microscope, bright field; (**b**) fluorescent microscope; (**c**–**e**) TEM, ultrathin sections. Magnification (bar): (**a**,**b**) 5 µ; (**c**) 1 µ, (**d**,**e**) 0.5 µ; (**a**) community of phototrophs of the chemocline zone of Black Kichier lake; (**b**–**e**) *Chloronema* sp.; (**c**) fluorescent microscope; (**a**,**d**,**e**) TEM, ultrathin section. tr—*Thiopedia rosea*; cn—*Chloronema* sp.; cy—cyanobacteria; tp—*Thiopedia rosea*; gv—gas vesicles; ch—chlorosome.

**Figure 15:**
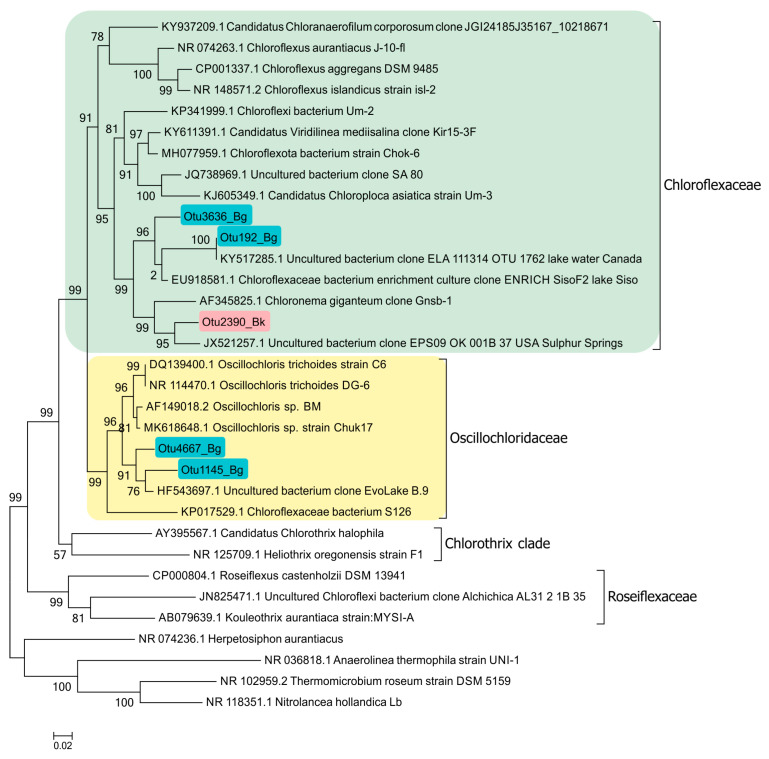
Phylogenetic position of planktonic *Chloroflexaceae* clones in the Black Kichier (Bk) and Big Kichier (Bg) lakes.

Planktonic filamentous phototrophic bacteria were revealed both by microscopy (Figure 14) and with molecular techniques (Figure 15). A phylogenetic analysis revealed the presence of *Chloronema* sp. in both Black (Otu2390_Bk) and Big Kichier (OTU 3636_Bg, OTU 192_Bg) [52].

In Lake Big Kichier, *Chloroflexota* related to *Oscillochloris* spp. (OTU 4667_Bg, OTU 1145_Bg) were also identified. This species was represented by straight or loosely spiral filaments surrounded by a mucous sheath. Ultrathin sections revealed extensive gas vacuoles filled with gas vesicles (cylindrical with pointed ends). The chlorosomes were located at the cell periphery, as shown in Figure 14.

## 4. Discussion

Lake Kichier, which has been a subject of microbiological research for half a century, is a classical karst lake with a high sulfide concentration in the hypolimnion. Our data confirmed the stability of its hydrochemical regime and the stability of the APB species composition in the chemocline zone of the Black and Big Kichier lakes.

The specific approach we used for APB identification was based on a combination of molecular diagnostic techniques with the results of light and electron microscopy of the water samples. Most works on microbial biodiversity rely on the computer processing of the sequencing data, with only the OTUs with relative abundance above 1% being considered. This approach disregarded the minor components of the communities. Moreover, uncultured ABP species belong to this minor fraction. We analyzed the OTUs of both the dominant and the minor groups of anoxygenic phototrophs revealed by the high-throughput sequencing of the 16S rRNA gene fragments. This approach made it possible to identify the dominant APB species in Lake Kichier chemocline and to obtain the DNA sequences of unknown APBs, among which certainly are those easily discernible by microscopy, including *Ancalochloris perfilievii* and other morphotypes of prosthecate GSB. As can be seen from the case of *Prosthecochloris aestuarii*, apart from prosthecate bacteria, organisms without cellular projections may belong to the genus *Prosthecochloris*. The projections may have diverse ecological functions, including energetic (additional chlorosomes are formed in prosthecae) and communicative ones (as organelles providing for close contact with the partner cells in syntrophic associations) [53]. Since prosthecae formation in GSB probably does not involve a significant genetic rearrangement, it may be expected that *Ancalochloris perfilievii* and other prosthecate bacteria of Lake Kichier are in fact unidentified species of the known genus *Chlorobium*. It should be noted that the cells of *Chlorobium clathratiforme* (previously named *Pelodictyon clathratiforme*) produce prosthecae-like pointed projections when forming their netlike microcolonies.

Our work was carried out when cyanobacteria *Planktothrix pseudoagarhdii*, which caused the summer bloom in Lake Kichier, were withering away. This bloom was responsible for the low values of bacterial photosynthesis. The second most abundant cyanobacteria were unicellular members of the genera *Cyanobium* and *Sinechococcus*, which were the most numerous in the mixolimnion, in the zone of the maximal oxygenic photosynthesis. Unexpectedly, the highest abundance of filamentous cyanobacteria was found in the chemocline zone near the GSB plate. No data are available in the literature concerning the ability of *Planktothrix* cyanobacteria to carry out anoxygenic photosynthesis with sulfide as the electron donor. It may therefore be suggested that GSB were responsible for sulfide oxidation in the Black and Big Kichier lakes.

A comparison of the composition of anoxygenic phototrophs identified in Lake Kichier by molecular techniques to the species identified previously (in the 1965–2007 period) based on their phenotypic characteristics revealed only insignificant differences. The sequencing of the 16S rRNA gene fragments confirmed the predominance of free-living GSB *Chlorobium clathratiforme* (green morphotype) and the presence of *Chlorochromatium magnum* and *Pelochromatium roseum*, as well as of the PSB *Chromatium okenii* and *Thiodiction bacillosus*.

It should be noted that while planktonic *Choroflexaceae* have been detected in a number of desalinated stratified lakes, their phylogenetic position is insufficiently studied. The absence of pure cultures of freshwater *Chloroflexota*, apart from *Oscillolochris trichoides* [54], is one of the reasons. The filamentous bacteria of the genera *Chloronema* and *Oscillochloris* identified in the Lake Kichier chemocline by molecular techniques were represented by new species [52]. The clones OTU2390_Bk, OTU3636_Bg, and OTU192_Bg, together with the clone “*Chloronema giganteum*” Gnsb-1, form the cluster comprising bacteria using the hydroxypropionate pathway of CO_2_ assimilation. At the same time, members of the clone OTU4667_Bg, as well as other known *Oscillochloris* isolates, form a cluster of photoautotrophic bacteria binding CO_2_ via the Calvin cycle. Other clones of uncultured phototrophic *Chloroflexota* were also revealed.

It may be concluded that the molecular identification of many APB species in the environment is presently impossible due to the absence of genomic data associated with well-known bacterial morphotypes. Our study proposes a comprehensive approach to the analysis of microbial diversity, implying a combination of up-to-date molecular diagnostic techniques with the data of light and electron microscopy and providing the guidelines for future taxonomic research.

## Figures and Tables

**Table 1:** Species identification of GSB from the chemocline zone of the Black (Bk) and Big Kichier (Bg) lakes based on analysis of the 16S rRNA gene fragments. The depth of maximal abundance is given in parentheses.

Bk OTU	Bg OTU	Identified Species	% Similarity	Depth of the Highest OTU Abundance
5	10	*Chlorobium clathratiforme*	99.25	Bk-7.47 (3.5 m); Bg-6.86 (4.5 m)
275	268	*Chlorobium* sp.	96.24 similarity to *Chlorobium clathratiforme*	Bk-0.05 (4 m) Bg-0.65 (4.5 m)
	9526			Bg-0.01 (5.0 m)
	937	*Chlorobaculum thiosulfatophilum*	98.5	Bg-0.02 (4.5 m)
188	398	*Chlorobiota*	*Chlorobium luteolum* 95.47(Bk); 97.37 (Bg)	Bk-0.32 (4.0 m) Bg-0.05 (4.0 m)
	8111	*Chlorobiota*	*Chlorobium limicola* 95.86	Bg-0.02 (4.0 m)
121	54	*Chlorochromatium magnum*	99.2	Bk-0.63 (3.5 m) Bg-0.87 (4.5 m)
798	533	*Pelochromatium roseum*	99.2	Bk-0.05 (4.0 m) Bg-0.35 (4.5 m)

## Data Availability

Raw 16S sequences can be found on the NCBI BioProject with the accession number PRJNA1007459.

## Supplementary Materials

The following supporting information can be downloaded at https://www.mdpi.com/article/10.3390/microorganisms12010013/s1, Figure S1. Location of the lakes Black Kichier and Big Kichier (Mari El Republic); Figure S2. Absorption spectra of the cells from the chemocline zone (a) and of ethanol extracts of the cells collected at 3.5 m in Lake Black Kichier and concentrated by filtration (b).

## References

[1] Boehrer B., Schultze M. (2008). Stratification of lakes. Rev. Geophys..

[2] Zadereev E.S., Gulati R.D., Camacho A. (2017). Biological and Ecological Features, Trophic Structure and Energy Flow in Meromictic Lakes. Mediterranean-Type Ecosystems.

[3] Mori Y., Kataoka T., Okamura T., Kondo R. (2013). Dominance of green sulfur bacteria in the chemocline of the meromictic Lake Suigetsu, Japan, as revealed by dissimilatory sulfite reductase gene analysis. Arch. Microbiol..

[4] Van Gemerden H., Mass J., Blankenship R.E., Madigan M.T., Bauer C.E. (1995). Ecology of phototrophic sulfur bacteria. Anoxygenic Photosynthetic Bacteria.

[5] Bolhuis H., Cretoiu M.S., Stal L.J. (2014). Molecular ecology of microbial mats. FEMS Microbiol. Ecol..

[6] Crowe S.A., Jones C.A., Katsev S., Magen C., O’Neill A.H., Sturm A., Canfield D.E., Haffner G.D., Mucci A., Sundby B. (2008). Photoferrotrophs thrive in an Archean Ocean analogue. Proc. Natl. Acad. Sci. USA.

[7] Poulton S., Canfield D. (2011). Ferruginous Conditions: A Dominant Feature of the Ocean through Earth’s History. Elements.

[8] Saini J., Hassler C., Cable R., Fourquez M., Danza F., Roman S., Tonolla M., Storelli N., Jacquet S., Zdobnov E. (2022). Bacterial, phytoplankton, and viral distributions and their biogeochemical contexts in meromictic Lake Cadagno offer insights into the Proterozoic Ocean microbial loop. MBio.

[9] Thiel V., Tank M., Bryant D. (2018). Diversity of chlorophototrophic bacteria revealed in the omics era. Annu. Rev. Plant Biol..

[10] Imhoff J.F., Hallenbeck P.C. (2017). Anoxygenic phototrophic bacteria from extreme environments. Modern Topics in the Phototrophic Prokaryotes: Environmental and Applied Aspects.

[11] Danza F., Ravasi D., Storelli N., Roman S., Lüdin S., Bueche M., Tonolla M. (2018). Bacterial diversity in the water column of meromictic Lake Cadagno and evidence for seasonal dynamics. PLoS ONE.

[12] Di Nezio F., Beney C., Roman S., Danza F., Buetti-Dinh A., Tonolla M., Storelli N. (2021). Anoxygenic photo- and chemo-synthesis of phototrophic sulfur bacteria from an alpine meromictic lake. FEMS Microbiol. Ecol..

[13] Linz A.M., He S., Stevens L.R., Anantharaman K., Rohwer R.R., Malmstrom R.R., Bertilsson S., McMahon K.D. (2018). Freshwater carbon and nutrient cycles revealed through reconstructed population genomes. PeerJ.

[14] Mehrshad M., Salcher M.M., Okazaki Y., Nakano S.I., Šimek K., Andrei A.S., Ghai R. (2018). Hidden in plain sight—Highly abundant and diverse planktonic freshwater Chloroflexi. Microbiome.

[15] Buck M., Garcia S.L., Fernandez L., Martin G., Martinez-Rodriguez G.A., Saarenheimo J., Zopfi J., Bertilsson S., Peura S. (2021). Comprehensive dataset of shotgun metagenomes from oxygen stratified freshwater lakes and ponds. Sci. Data.

[16] Cabello-Yeves P.J., Picazo A., Roda-Garcia J.J., Rodriguez-Valera F., Camacho A. (2023). Vertical niche occupation and potential metabolic interplay of microbial consortia in a deeply stratified meromictic model lake. Limnol. Oceanogr..

[17] Gorbunov M.Y., Umanskaya M.V. (2020). Karst Lakes of Mari Chodra National Park: Stratification and vertical distribution of phototrophic plankton. IOP Conf. Ser. Earth Environ. Sci..

[18] Ruzsky M. (1916). Limnological studies in the middle Volga region. Lakes of the Northwestern Part of the Kazan Province.

[19] Kuznetsov S.I. (1952). The Role of Microorganisms in the Cycling of Substances in Lakes.

[20] Kuznezow S.I., Gorlenko V.M. (1973). Limnologische und Mikrobiologische Eigenschaften von Karstseen der ASR Mari. Arch. Hydrobiol..

[21] Gorlenko V.M., Dubinina G.A., Kuznetsov S.I. (1983). The Ecology of Aquatic Microorganisms.

[22] Kellenberger E., Ryter A., Séchaud J. (1958). Electron microscope study of DNA-containing plasms. II. Vegetative and mature phage DNA as compared with normal bacterial nucleoids in different physiological states. J. Cell Biol..

[23] Reynolds E.S. (1963). The use of lead citrate stain at high pH in electron microscopy. J. Cell Biol..

[24] Overmann J., Tilzer M.M. (1989). Control of primary productivity and the significance of photosynthetic bacteria in a meromictic kettle lake Mittlerer Buchensee, West-Germany. Aquat. Sci..

[25] Sorokin Y.I., Kadota H. (1972). Techniques for the assessment of microbial production and decomposition in fresh waters. IBP Handbook No 23.

[26] Savvichev A.S., Kokryatskaya N.M., Zabelina S.A., Rusanov I.I., Zakharova E.E., Veslopolova E.F., Lunina O.N., Patutina E.O., Bumazhkin B.K., Gruzdev D.S. (2017). Microbial Processes of the carbon and sulfur cycles in an ice-covered, iron-rich neromictic Lake Svetloe (Arkhangelsk Region, Russia). Environ. Microbiol..

[27] Magoć T., Salzberg S.L. (2011). FLASH: Fast length adjustment of short reads to improve genome assemblies. Bioinformatics.

[28] Edgar R.C. (2010). Search and clustering orders of magnitude faster than BLAST. Bioinformatics.

[29] Pruesse E., Quast C., Knittel K., Fuchs B.M., Ludwig W., Peplies J., Glöckner F.O. (2007). SILVA: A comprehensive online resource for quality checked and aligned ribosomal RNA sequence data compatible with ARB. Nucleic Acids Res..

[30] Gich F., Borrego C.M., Martinez-Planells A., Garcia-Gil J., Garab G. (1998). Adaptation of the photosynthetic antenna of *BChl* d-containing green sulfur bacteria to low light intensities. Photosynthesis: Mechanisms and Effects.

[31] Dubinina G.A., Gorlenko V.M. (1975). New filamentous photosynthesizing green bacteria with gas vacuoles. Mikrobiologiia.

[32] Komárek J., Kopecký J., Cepák V. (1999). Generic characters of the simplest cyanoprokaryotes Cyanobium, Cyanobacterium and Synechococcus. Cryptogam. Algol..

[33] D’alelo D., Salmaso N. (2011). Occurrence of an uncommon Planktothrix (*Cyanoprokaryota*, *Oscillatoriales*) in a deep lake south of the Alps. Phycologia.

[34] Abella C.A., Garcia-Gil L.J., Olson J.M., Ormerod J.G., Amesz J., Stackebrandt E., Trüper H.G. (1988). Dial migration as a mechanism for enrichment of natural populations of branching species of Pelodictyon. Green Photosynthetic Bacteria.

[35] Overmann J., Pfennig N. (1989). *Pelodictyon phaeoclathratifovme* sp. nov., a new brown-colored member of the Chlorobiaceae forming net-like colonies. Arch. Microbiol..

[36] Gich F.B., Garcia-Gil L.J., Overmann J. (2001). Previously unknown and phylogenetically diverse members of the green nonsulfur bacteria are indigenous to freshwater lakes. Arch. Microbiol..

[37] Gorlenko V.M., Lebedeva E.V. (1971). New green bacteria with the out-growths. Mikrobiologiya.

[38] Anagnostides K., Overbeck J. (1966). Methanoxydierer und hypolimnisehe Schwefelbakterien. Ber. Deut. Bodenk. Ges..

[39] Caldwell D.E., Tiedje J.M. (1975). A morphological study of anaerobic bacteria from the hypolimnia of two Michigan lakes. Can. J. Microbiol..

[40] Caldwell D.E., Tiedje J.M. (1975). The structure of anaerobic bacterial communities in the hypolimnia of several Michigan lakes. Can. J. Microbiol..

[41] Skuia H. (1956). Taxonomische und biologische Studien iiber das Phytoplankton schwedischer Binnengewasser. Nova Acta Reg. Soc. Sci. Upsal..

[42] Caldwell D.E., Overbeck J. (2008). The Planktonic Microflora of Lakes. CRC Crit. Rev. Microbiol..

[43] Overmann J., Schubert K. (2002). Phototrophic consortia: Model systems of symbiotic relationships between prokaryotes. Arch. Microbiol..

[44] Glazer J., Overmann J. (2004). Biogeography, evolution and diversity of epibionts in phototrophic consortiums. Appl. Environ. Microbiol..

[45] Kanzler B.E., Pfannes K.R., Vogl K., Overmann J. (2005). Molecular characterization of a non-photosynthetic partner bacterium in the “Chlorochromatium aggregatum” consortium. Appl. Environ. Microbiol..

[46] Pfannes K.R., Vogl K., Overmann J. (2007). Heterotrophic symbionts of phototrophic consortia: Members of a novel diverse cluster of Betaproteobacteria characterized by a tandem rrn operon structure. Environ. Microbiol..

[47] Vogl K., Venter R., Dressen M., Schlickenrieder M., Plescher M., Eyhaker L., Overmann J. (2008). Identification and analysis of four candidate symbiosis genes from “Chlorochromatium aggregatum”, a highly developed bacterial symbiosis. Environ. Microbiol..

[48] Vila X., Abella C.A., Figueras J.B., Hurley J.P. (1998). Vertical models of phototrophic bacterial distribution in the metalimnetic microbial communities of several freshwater North-American kettle lakes. FEMS Microbiol. Ecol..

[49] Peduzzi S., Storelli N., Welsh A., Peduzzi R., Hahn D., Perret X., Tonolla M. (2012). *Candidatus* “Thiodictyon syntrophicum”, sp. nov., a new purple sulfur bacterium isolated from the chemocline of Lake Cadagno forming aggregates and specific associations with *Desulfocapsa* sp.. Syst. Appl. Microbiol..

[50] Luedin S.M., Liechti N., Cox R.P., Danza F., Frigaard N.U., Posth N.R., Pothier J.F., Roman S., Storelli N., Wittwer M. (2019). Draft genome sequence of *Chromatium okenii* isolated from the stratified Alpine Lake Cadagno. Sci. Rep..

[51] Eichler B., Pfennig N. (1991). Isolation and characteristics of Thiopedia rosea (neotype). Arch. Microbiol..

[52] Abella C.A., Garcia-Gil L.J. (1990). Microbial ecology of planktonic filamentous phototrophic bacteria in holomictic freshwater lakes. The Dynamics and Use of Lacustrine Ecosystems: Proceedings of the 40-Year Jubilee Symposium of the Finnish Limnological Society, Helsinki, Finland, 6–10 August 1990.

[53] Ha P., Lindemann S., Shi L., Dohnalkova A., Fredrickson J., Madigan M., Beyena H. (2017). Syntrophic anaerobic photosynthesis via direct interspecies electron transfer. Nat. Commun..

[54] Keppen O.I., Tourova T.P., Kuznetsov B.B., Ivanovsky R.N., Gorlenko V.M. (2000). Proposal of *Oscillochloridaceae* fam. nov. on the basis of aphylogenetic analysis of the filamentous anoxygenic phototrophic bacteria, and emended description of Oscillochloris and Oscillochloris trichoides in comparison with further new isolates. Int. J. Syst. Evol. Microbiol..

