# Supplementary figures and images for: A Novel View of the Diversity of Anoxygenic Phototrophic Bacteria Inhabiting the Chemocline of Meromictic Karst Lakes
Source: Microorganisms. 2023 Dec 20;12(1):13. doi: 10.3390/microorganisms12010013 (PMC10820006; doi:10.3390/microorganisms12010013)

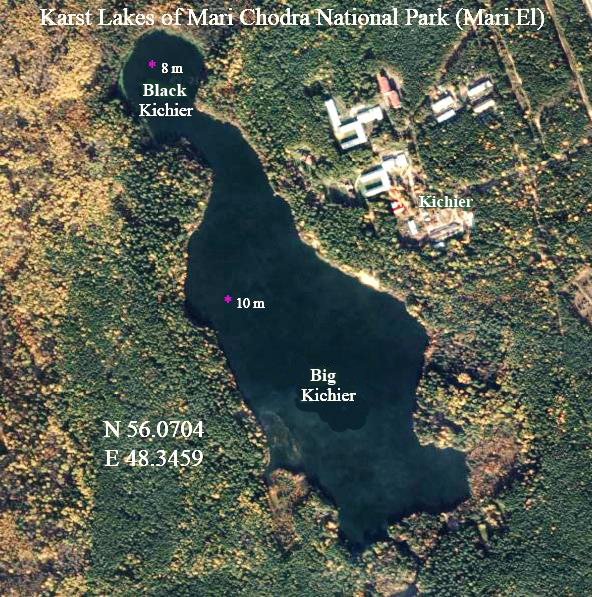

Supplement: Supplementary file 1 [file microorganisms-12-00013-s001.zip › Supplementary Figure S1.jpg]

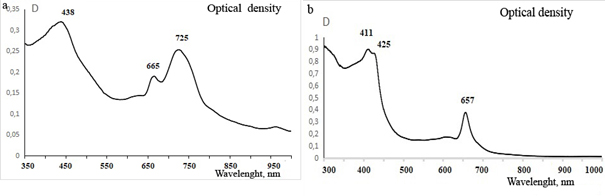

Supplement: Supplementary file 1 [file microorganisms-12-00013-s001.zip › Supplementary Figure S2.jpg]
